# Interesting Biochemistries in the Structure and Function of Bacterial Effectors

**DOI:** 10.3389/fcimb.2021.608860

**Published:** 2021-02-24

**Authors:** Hazel Mak, Teresa L. M. Thurston

**Affiliations:** MRC Centre for Molecular Bacteriology and Infection, Imperial College London, London, United Kingdom

**Keywords:** bacterial effectors, secretion systems, structure-function, pathogenesis, protein organization, host-pathogen

## Abstract

Bacterial effector proteins, delivered into host cells by specialized multiprotein secretion systems, are a key mediator of bacterial pathogenesis. Following delivery, they modulate a range of host cellular processes and functions. Strong selective pressures have resulted in bacterial effectors evolving unique structures that can mimic host protein biochemical activity or enable novel and distinct biochemistries. Despite the protein structure-function paradigm, effectors from different bacterial species that share biochemical activities, such as the conjugation of ubiquitin to a substrate, do not necessarily share structural or sequence homology to each other or the eukaryotic proteins that carry out the same function. Furthermore, some bacterial effectors have evolved structural variations to known protein folds which enable different or additional biochemical and physiological functions. Despite the overall low occurrence of intrinsically disordered proteins or regions in prokaryotic proteomes compared to eukaryotes proteomes, bacterial effectors appear to have adopted intrinsically disordered regions that mimic the disordered regions of eukaryotic signaling proteins. In this review, we explore examples of the diverse biochemical properties found in bacterial effectors that enable effector-mediated interference of eukaryotic signaling pathways and ultimately support pathogenesis. Despite challenges in the structural and functional characterisation of effectors, recent progress has been made in understanding the often unusual and fascinating ways in which these virulence factors promote pathogenesis. Nevertheless, continued work is essential to reveal the array of remarkable activities displayed by effectors.

## Introduction

The pathogenesis of many Gram-negative bacteria is highly dependent on specialized multiprotein machines that deliver a repertoire of bacterial effectors in a spatiotemporally coordinated manner into the host cell, where they modulate a range of eukaryotic cellular processes. These specialized multiprotein machines are known as secretion systems. Of the seven known secretion systems found in Gram-negative bacteria, the direct delivery of effectors across a host cell membrane can only be achieved by the type III, type IV, and type VI secretion systems (T3SS, T4SS, and T6SS respectively) (Galán and Waksman, 2018). The type VII secretion system present in Gram-positive bacteria and mycobacteria will not be discussed here. Through horizontal gene transfer (HGT) and selective pressure from the host(s), pathogenic bacteria have acquired bacterial effectors, mainly encoded on pathogenicity islands and virulence plasmids. The function of bacterial effectors is varied but broadly they promote bacterial invasion and colonisation of host cells, as well as bacterial survival, growth, and replication. Other key effector functions include modulating host immune signaling and establishing a bacteria-beneficial niche within the host. This alters the relationship between pathogen and host, with bacterial effectors reprogramming complex eukaryotic processes to promote a parasitic relationship, where the pathogen is supported by its host. Parasitic relationships result in injury, disease, and potentially death of the host. However, host cells detect pathogen-associated molecular patterns (PAMPs) from invading organisms, and this activates a cascade of pro-inflammatory signaling and defence mechanisms that can protect the host from the invading pathogen (Janeway, 1989). In addition, effector-triggered immunity (ETI), which is well characterized in plant cells, is now reported in metazoans and refers to the initiation of a protective immune response upon detection of bacterial toxins, secreted proteins or the detection of their activities (Lopes Fischer et al., 2020). In this way, ETI provides another layer of immune defence that detects pathogen manipulation of key cellular processes, including the subversion of host immune responses. This refocuses the relationship from being parasitic to one where the host gains immunity, in order to resist and clear the pathogen. As a result of adaption and the evolution of this bacteria-host relationship, different bacterial species have acquired specific repertoires of effectors. Nevertheless, common themes, related to effector structure and function, exist among effectors from diverse pathogens.

In general, bacterial proteins that are not secreted, have an individual biochemical activity associated to a physiologically relevant function and structure within the bacteria. This follows the structure-function paradigm, where the function of a protein is directly related to its three-dimensional structure. However, effectors are distinct from other bacterial proteins, as they primarily function and exert their biochemical activity within the target cell, rather than within the bacteria. Of the different secretion systems that deliver bacterial proteins across a mammalian cell membrane, the T6SS is the most recently identified and still remains poorly described in terms of effector delivery and the action of T6SS effectors within eukaryotic host cells. Therefore, we will not discuss T6SS effectors further here. Prior to effector delivery through the multiprotein T3SS, many but not all T3SS effectors are chaperoned to the base of the secretion system. Here, they are secreted in an ATP-dependent, unfolded or partially folded and inactive state, which allows passage through the narrow secretion system tunnel (Radics et al., 2014; Dohlich et al., 2014). Once delivered into a eukaryotic host cell, the folding of effectors into their active conformation may or may not require host proteins and additional, host-mediated, post-translational modifications. This supports a hypothesis whereby T3SS effectors primarily function within host cells. However, there is at least one example describing effector catalytic activity within the pathogen (Qaidi et al., 2020). The T3SS effector and *N*-acetylglucosamine transferase, NleB, from pathogenic *Escherichia coli*, modifies bacterial glutathione synthetase (GshB) to promote GshB activity and bacterial survival to oxidative stress (Qaidi et al., 2020). Given this unexpected observation, further experiments should investigate how widespread this phenomenon is.

T4SSs are versatile systems capable of secreting protein and DNA into target cells that include other bacteria and eukaryotic cells. Relevant to this review is the delivery of proteins through the T4SS into host eukaryotic cells from pathogens such as *Legionella pneumophila* and *Helicobacter pylori*. The translocation of T4SS effectors is similar to T3SS effectors; most effectors are unfolded or partially folded and in complex with a chaperone for secretion (Costa et al., 2015; Sgro et al., 2019). There are also examples of folded proteins that need to be unfolded for T4SS translocation (Trokter and Waksman, 2018).

Secretion systems have evolved to deliver bacterial effectors in a spatiotemporally regulated and coordinated manner (Selkrig et al., 2020), which enables effectors to work in concert with each other. For example, the *Salmonella* T3SS effectors SseF and SseG, function together to anchor *Salmonella*-containing vacuoles (SCV) to the Golgi Network (Yu et al., 2016) and the global mapping of *Salmonella*-host protein-protein interactions revealed that SseJ and SseL collaborate in order to redirect cholesterol to the SCV (Walch et al., 2020). Effectors working in opposition to each other have also been described, for example, *Legionella* LubX targets the bacterial effector SidH for degradation *via* the host proteasome in the later stages of infection and this finding gave rise to the term “meta-effectors”, or “effectors of effectors” (Kubori et al., 2010; Urbanus et al., 2016). Another key difference between bacterial proteins and secretion system effectors is that many effectors have more than one host cellular target and hence may have multiple biochemical activities and biological functions (Galán, 2009; Walch et al., 2020).

The relatively low concentration of many effectors within host cells is likely to drive the evolution of enzymatic activities, yet some effectors appear to function as adaptors. Many effectors that lack their own enzymatic activity function by recruiting and redirecting host enzymes to indirectly modify target protein(s) and modulate host cell signaling (Ohlson et al., 2008; Bayer-Santos et al., 2016; Panagi et al., 2020). Like eukaryotes, prokaryotes exploit the use of post translational modifications (PTMs) to increase the functional diversity of their proteome in a dynamic way. However, the repertoire of PTMs is divergent to that found in eukaryotes. For example, prokaryotes lack the full array of enzymes required for the conjugation of ubiquitin to target proteins. Nevertheless, bacteria have evolved to exploit host machinery to carry out ubiquitination *via* bacterial E3 ligase effectors as well as various other PTMs that are not required for the regulation of bacterial physiology.

In this review, we will examine common themes of effectors from diverse bacterial species in terms of structure and biochemical activity. We will take examples from several pathogens that utilize type III and type IV secretion systems yet acknowledge that we are unable to review the vast array of effector-mediated functions and biochemical activities. The first group of effectors we will consider are those that have adopted structural similarities to eukaryotic proteins that enable them to mimic the biochemical activities of host proteins. For example, effectors that act as proteases, phosphatases, kinases, glycosylases, and more, have been described. Other bacterial effectors have unique structures that lack homology to our current knowledge of eukaryotic proteins and have interesting biochemical activities that perform unique and alternative PTMs and functions in comparison to those exhibited by normal eukaryotic processes. The final structural property of bacterial effectors we will consider is the occurrence of intrinsically disordered regions (IDRs). In general, prokaryotic proteomes show a low degree of IDRs when compared to eukaryotes (Dunker et al., 2000). However, bacterial effectors seem comparatively enriched with IDRs that mimic those found in mammalian proteins (Iakoucheva et al., 2002; Marín et al., 2012). As described below, these IDRs are likely to mediate specific host-pathogen protein-protein interactions. Together, these structural aspects enhance the potency of the effector. Enzymatic mimicry and novel biochemistry are unlikely to be directly inhibited or reversed by the host and precise protein-protein interactions ensure a high degree of specificity for effector activity.

We will end our review by highlighting some of the current challenges in characterising the structure and function of effectors in different bacterial species as well as the advances in experimental techniques that may be used to improve our knowledge and characterization of bacterial effectors. Understanding the structures and functions of diverse effectors improves our understanding of the mechanisms that drive bacterial pathogenesis. Furthermore, uncovering unique biochemical mechanisms, which appear to be absent from normal host cell biology, provides potentially new targets for the development of antimicrobials that will not interfere with host biochemistry.

## Structured “Ordered” Bacterial Effectors Mimicking Host Protein Function

A large majority of characterized bacterial effectors are well-defined, structured, and ordered proteins with a stable, fixed three-dimensional structure that influences the effector function. Within this group of structured effectors, there are numerous examples where effectors have evolved to mimic the biochemical activity or structural properties of host cell proteins without significant sequence or structural homology to any particular host protein. The use of eukaryotic-like domains to mimic endogenous cellular proteins could represent a selective advantage for bacteria as the activity of these effectors might not be directly inhibited by host proteins and the diversification in protein structure might also result in additional physiological functions that enhance the virulence potential of the effector. In addition, bacterial effectors that fine-tune host cell processes using eukaryotic-like biochemistry might promote the silent manipulation of host cell signaling without triggering ETI. Finally, it is interesting to consider that some effectors have also evolved that mimic eukaryotic-like protein biochemistry but act towards other bacterial proteins as well as host proteins. The *Legionella* meta-effector and E3 ubiquitin ligase, LubX, acts to spatiotemporally regulate the activity of the effector SidH (Kubori et al., 2010). LubX contains two U-box domains, one that serves as an E2-binding site and a second U-box that functions as a substrate binding site. In this way, LubX has evolved to exploit the host ubiquitination machinery and proteasome in order to regulate one of its own effectors within host cells (Kubori et al., 2010).

**Figure 1:**
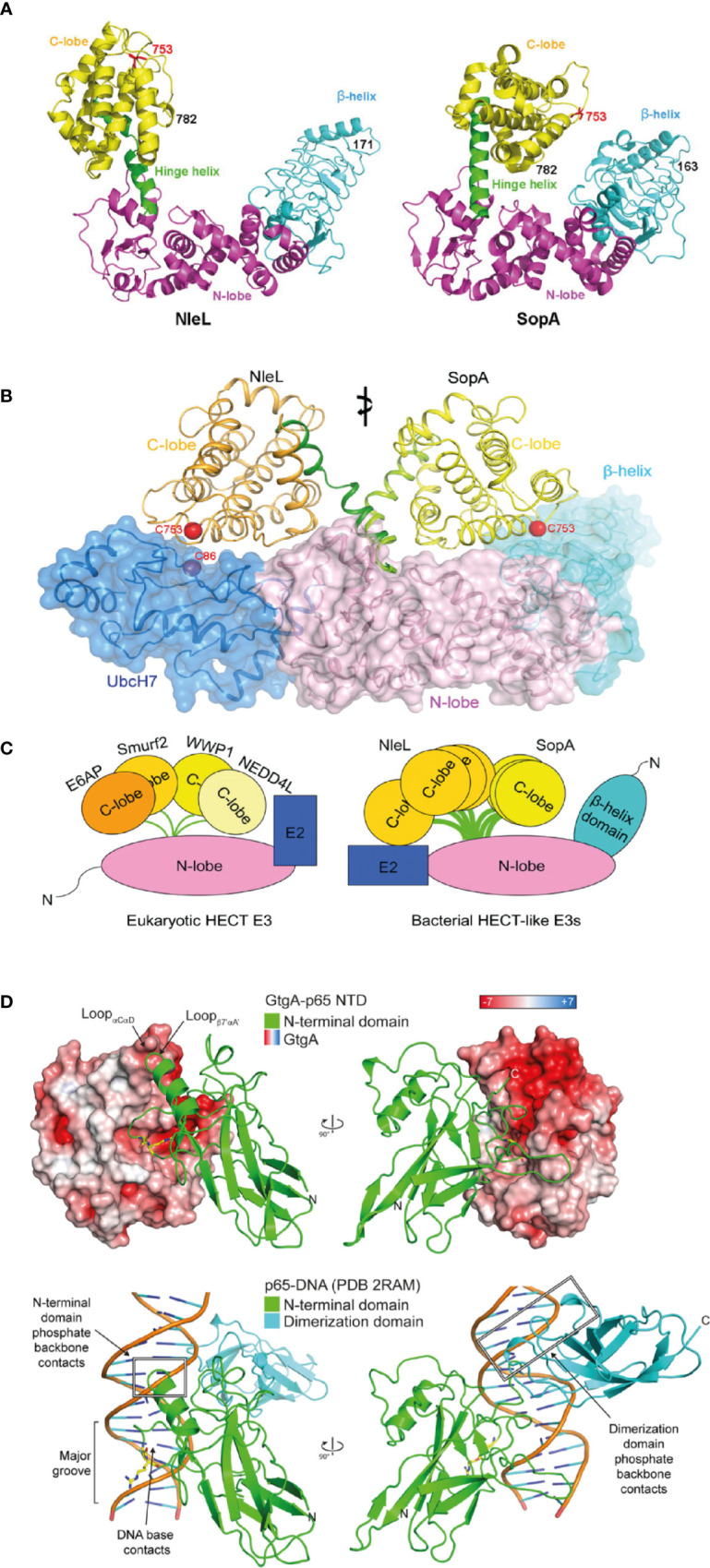
Structured bacterial effectors mimicking host cell proteins. **(A)** Structural comparison between Enterohemorrhagic *E. coli* (EHEC) effector NleL (residue 170-782) and *Salmonella* effector SopA (residue 163-782) in ribbon cartoon representation. Structures consist of the N-lobe (magenta), the C-lobe (yellow) and the β-helix domain (cyan). The catalytic cysteine (Cys) residue 753 are labelled in red. The hinge helix is labelled and shown in green (taken from Lin et al., 2011). **(B)** Structural superposition of two bacterial HECT-like E3 ligases, SopA from *Salmonella* and NleL from EHEC, bound to human E2 protein UbcH7 (shown in dark blue). The E3 ligase N-lobes and β-helix domains are shown in pink and light blue respectively, as ribbon representation and transparent surface. The C-lobe is structurally flexible and shown in orange for NleL and in yellow for SopA. The catalytic cysteine (Cys) residues are shown in red (taken from Lin et al., 2012). **(C)** Schematic of structural comparison between eukaryotic HECT E3 ligases and bacterial HECT-like E3 ligases. Eukaryotic HECT E3 ligases include E6AP, Smurf2, WWP1, and NEDD4L. Bacterial HECT-like E3 ligases include SopA from *Salmonella* and NleL from EHEC. Structural flexibility is shown in the C-lobe of NleL and SopA (taken from Lin et al., 2012). **(D)** Structural mimicry of DNA by *Salmonella* effector GtgA. Top structure shows GtgA in complex with the N-terminal domain (NTD) of p65. Bottom structure shows DNA in complex with the NTD and the dimerization domain of p65. GtgA is shown in surface representation and coloured according to its electrostatic surface potential (red is negative; white is neutral; blue is positive). The NTD of p65 is shown in green and the dimerization domain of p65 is shown in cyan. The cleavage site residues in p65 (Gly-40/Arg-41) are shown with yellow sticks (taken from Jennings et al., 2018).

The *Salmonella* T3SS effector SopA and the Enterohemorrhagic *E. coli* (EHEC) effector NleL represent homologous proteins that both exhibit ubiquitin ligase activity. SopA and NleL, which do not share any sequence homology, contain some structural similarities to each other and the host cell protein domain, eukaryotic E3 ubiquitin ligase homologous to E6-AP carboxy terminus (HECT), which mediates the addition of ubiquitin on to target proteins (Zhang et al., 2006; Lin et al., 2011). Crystal structures of both SopA and NleL show the distinguishing bi-lobal structure of HECT domains (Lin et al., 2012) (**Figure 1A**). Both SopA and NleL also contain a conserved C-terminal region cysteine residue that is required to form the thioester-linked intermediate prior to ubiquitin transfer to the substrate. The N-lobe contains the E2-binding site and is attached to a structurally flexible C-lobe in SopA and NleL (**Figures 1B, C**). Similar to the HECT domain, the structural flexibility between N- and C-lobes most likely enables SopA and NleL to interact with E2 ubiquitin-conjugating enzymes and to ubiquitinate target host proteins. Through molecular mimicry, both SopA and NleL bind the canonical surface of the E2 UbcH7, hijacking the host ubiquitination machinery despite showing little similarity to the E2-interacting surface of eukaryotic HECT E3 ligases (Lin et al., 2012). In comparison to the HECT domain, there is an additional N-terminal β-helix domain in SopA and NleL (**Figure 1C**). While functionally uncharacterized, this β-helix domain may act as a substrate binding site (Fiskin et al., 2017). The lack of sequence similarity between SopA and NleL results in differing molecular surfaces which might explain the functional differences observed (Diao et al., 2008; Lin et al., 2012). SopA modulates *Salmonella*-induced intestinal inflammation and stimulation of transepithelial migration of polymorphonuclear leucocytes (Lin et al., 2011; Kamanova et al., 2016). SopA appears to function through interaction with and ubiquitination of TRIM56 and TRIM65, however the precise molecular mechanism remains controversial (Kamanova et al., 2016; Fiskin et al., 2017). In contrast, NleL inhibits formation of actin membrane protrusions, called pedestals, on the surface of host cells by the EHEC effector Tir by an unknown mechanism (Piscatelli et al., 2011). Alternatively, NleL might promote EHEC adherence by targeting host c-Jun NH2-terminal kinases (JNKs) (Sheng et al., 2017) and overexpressed NleL inhibits NF-κB signaling (Sheng et al., 2020). This highlights that although effectors might share structural and biochemical similarities, unique physiological functions are likely to have evolved.

Another example of effectors exploiting molecular mimicry is the family of effector zinc metalloproteases. This family consists of GtgA, GogA, and PipA from *Salmonella enterica*, NleC, and NleD from enteropathogenic *E. coli* and EHEC and RipAX2 from *Ralstonia solanacearum*, which all contain a conserved short metal binding HEXXH motif essential for catalytic activity. Although these proteins do not share significant sequence homology to known host zinc metalloproteases, they structurally retain the catalytic core of the Zincin superfamily (Jennings et al., 2018). Due to the diverse sequence homology found within the family, its members target different host proteins. NleD directly cleaves mitogen-activated protein kinases (MAPKs), JNK, and p38, in the flexible activation loop, thereby inhibiting activator protein-1 (AP-1)-dependent gene transcription and the JNK-dependent apoptosis (Baruch et al., 2011; Gur-Arie et al., 2020). In contrast, GtgA, GogA, PipA, and NleC specifically and directly cleave subunits of NF-κB to suppresses host pro-inflammatory immune responses (Baruch et al., 2011; Sun et al., 2016; Jennings et al., 2018) and NleC also cleaves and degrades the host acetyltransferase and transcriptional coactivator, p300 (Shames et al., 2011). Despite superficial similarity in targeting NF-κB subunits, GtgA, GogA, and PipA only cleave p65, RelB and cRel, whereas NleC can also hydrolyse p105/p50 and p100/p52 (Jennings et al., 2018). This molecular specificity arises from different cleavage sites, with GtgA, GogA, and PipA cleaving p65 between Gly40 and Arg41 (Sun et al., 2016). Arg41, in the P1’ position, is conserved in p65, RelB and cRel and is accommodated by a negatively charged pocket within GtgA, but p105/p50 and p100/p52 encode a proline at the corresponding residue which prevents cleavage (Jennings et al., 2018). In contrast, NleC cleaves p65 between residue Cys-38 and Glu-39 and the P1’ residue is conserved in all five NF-κB subunits. Despite these differences, both NleC and GtgA target NF-κB subunits through mimicry of the major groove of DNA (**Figure 1D**), which represents the normal binding target for nuclear NF-κB (Turco and Sousa, 2014; Jennings et al., 2018). Therefore, GtgA, GogA, PipA, and NleC show two forms of structural mimicry; first, functionally they act as zinc metalloproteases without sharing significant sequence homology to other known zinc metalloproteases and second, they mimic DNA in order to mediate substrate binding.

Although many bacterial effectors do not resemble eukaryotic host cell proteins in their overall structure, they might share short sequence motifs that are found in both eukaryotic proteins and effectors from different bacterial species. For example, a group of T3SS effectors that arose from convergent evolution share a conserved tryptophan (W)-xxx-glutamine (E) motif, which is found among effectors from diverse species and among TIR (Toll/Interleukin-1 receptor)-domain containing eukaryotic proteins (Felix et al., 2014). The WxxxE family of T3SS effectors include *Shigella* effectors IpgB1 and IpgB2, *Salmonella* effectors SifA and SifB (Alto et al., 2006), and EPEC and EHEC effectors Map (Kenny et al., 2002), EspM (Arbeloa et al., 2008), and EspT (Bulgin et al., 2009). In addition, despite not containing a WxxxE motif, *Salmonella* effectors SopE and SopE2, and BopE from *Burkholderia* share similar 3D structures to the WxxxE effectors and are therefore grouped into a larger family of effectors, known as the WxxxE effector and SopE-like family (Bulgin et al., 2010). Within this family of effectors, the WxxxE motif appears to have a structural role in maintaining the conformation of the putative catalytic loop, which mediates intrinsic guanine nucleotide exchange factor (GEF) activity towards Rho GTPases (Felix et al., 2014). Mechanistically, GDP to GTP exchange appears to mimic the “push and pull” mechanism exhibited by certain eukaryotic Rho GTPase GEFs. That is, interactions between the effector catalytic motif with the switch I and switch II regions on the target Rho GTPase lead to a conformational change that ejects GDP. Functionally, this effector-mediated manipulation of Rho GTPases controls host actin dynamics, with each effector showing specificity for different GTPases that mediate differential function (Bulgin et al., 2010). Interestingly, although SifA contains the conserved WxxxE motif in its C-terminal domain, SifA lacks the catalytic residues in the putative catalytic loop required for GEF activity, and does not show GEF activity *in vivo* (Ohlson et al., 2008). Instead, the N-terminal domain and C-terminal domain of SifA interact with protein partners independently, suggesting that SifA may have evolved from a GEF to an adaptor protein related to GTPase activity (Ohlson et al., 2008; Jennings et al., 2017). As in the above examples, the study of the WxxxE/SopE-like effectors illustrates how functional mimicry, in this case Rho GTPase GEF activity, is achieved without structural homology to host enzymes.

Of note, some bacterial effectors share modular sequence homology with diverse effectors and have more than one biochemical activity. For example, the *Salmonella* effector SptP contains two biochemical activities; the N-terminal domain contains a GTPase-activating protein (GAP) domain that is similar to YopE of *Yersinia* and ExoS of *Pseudomonas aeruginosa*, whereas the C-terminal domain shows sequence similarity to the protein tyrosine phosphatase YopH of *Yersinia* (Zhou and Galán, 2001). Both the GAP and tyrosine phosphatase activity contribute to SptP inhibiting Raf activation and the subsequent ERK MAPK signaling pathway (Lin et al., 2003). This dual activity is key in promoting proinflammatory cytokine release and dampens innate immune signaling and pathogen clearance in the host. Such dual activity is rare among non-secreted and non-virulent bacterial proteins.

In this section, we have highlighted examples whereby bacterial effectors with ordered structures mimic host cell protein biochemical activity and function but in the absence of significant sequence similarity. Next, we will consider effectors that mediate eukaryotic-like covalent modification through entirely novel protein folds as well as previously unseen post-translational modifications that have not been described in the study of eukaryotic biochemistry.

## Structured “Ordered” Bacterial Effectors With New Protein Folds and Biochemistry

In recent years, an array of distinct and novel biochemical mechanisms that are catalyzed by bacterial effectors, but seemingly not eukaryotic proteins, have been identified. This may be advantageous to the pathogen as the effectors and/or their modified host targets are less likely to be controlled by host feedback loops and/or regulatory proteins and evolution of host resistance mechanisms is likely to require significant time. These PTMs manipulate host cell signaling and cause detrimental downstream effects to host responses.

### Effector-Mediated Ubiquitination and Phosphorylation

**Figure 2:**
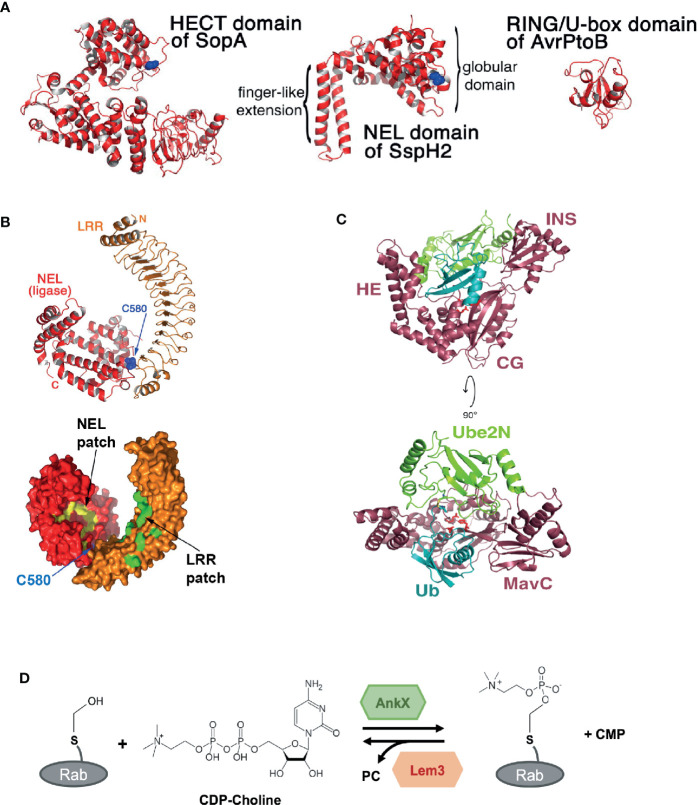
Novel structures and biochemical activities mediated by bacterial effectors. **(A)** Structural comparison between the Novel E3 Ligase (NEL) domain in *Salmonella* effector SspH2 with bacterial E3 ligases that mimic eukaryotic E3 ligase domains: the homologous to E6-AP carboxy terminus (HECT) domain in SopA from *Salmonella* and the Really Interesting New Gene (RING/U-box) domain in AvrPtoB from *Pseudomonas Syringae*. Catalytic cysteine residues are shown in blue (taken from Quezada et al., 2009). **(B)** Crystal structure of *Salmonella* effector SspH2 shown in ribbon representation (top) and molecular surface representation (bottom). The Novel E3 Ligase (NEL) domain is shown in red and the leucine-rich repeat (LRR) domain in orange. The catalytic cysteine residue in SspH2 (C580) is shown in blue. Hydrophobic patches are labelled and shown in yellow for the NEL domain and in green for the LRR domain (taken and adapted from Quezada et al., 2009). **(C)** Crystal structure of the *Legionella* effector MavC bound to E2 Ube2N-ubiquitin conjugate. MavC is shown in dark pink, Ube2N in green and ubiquitin (Ub) in blue. The domains of MavC are labelled: helical extension (HE), core globular domain (CG), and insertion domain (INS). The active site residues in MavC (C74, H231, and Q252) are shown as red sticks (taken from Puvar et al., 2020). **(D)** Phosphocholination and dephosphocholination of Rab GTPase protein by the *Legionella* effector AnkX and Lem3. AnkX catalyzes phosphocholination, the transfer of the phosphocholine moiety from cytidine diphosphate (CDP)-choline onto the hydroxyl group of a serine residue in certain Rab GTPase proteins. Lem3 catalyzes the dephosphocholination by removing the phosphocholine (PC) (adapted from Heller et al., 2015).

Protein ubiquitination is key in regulating many eukaryotic (but not prokaryotic) cellular processes. Interestingly however, bacteria have evolved different types of effector biochemistry that uniquely mediate, target and modify eukaryotic protein ubiquitination. The first example we will consider is the evolution of a family of “novel E3 ligases” (NELs) found among effectors that are structurally unique from mammalian E3 ligases (**Figure 2A**). This diversifies the repertoire of ubiquitin ligases that target host proteins, in addition to the bacterial E3 ligases mimicking eukaryotic E3 ligase domains (Ashida and Sasakawa, 2017). The NEL family contains members from at least six pathogenic bacteria, including *Salmonella* effectors SIrP (Bernal-Bayard and Ramos-Morales, 2009), SspH1 and SspH2 (Haraga and Miller, 2006; Quezada et al., 2009; Levin et al., 2010; Keszei et al., 2014), YopM of *Yersinia* (Soundararajan et al., 2011), and the IpaH effector family from *Shigella* (Singer et al., 2008). Although structurally unlike known eukaryotic E3 ligases, NELs show three key similarities that support ubiquitination. Similar to eukaryotic HECT E3 ligases, NELs contain a conserved catalytic cysteine residue that forms the ubiquitin thioester intermediate prior to ubiquitin transfer onto the substrate. NEL domains also contain a potential E2-interacting surface (Quezada et al., 2009), which enables these NEL effectors to bind to host E2 enzymes charged with ubiquitin, and compete with host E3 ligase proteins. Finally, representing a defining point of NEL family effectors is the presence of a canonical leucine-rich repeat (LRR) domain (**Figure 2B**) that interacts with the NEL domain to form an autoinhibitory fold. This prevents premature activation of the ligase, providing exquisite control of effector activity and might also prevent cellular toxicity induced by the NEL domain, which when expressed alone is toxic (Quezada et al., 2009; Chou et al., 2012; Keszei et al., 2014). This highly specific target bound activity might enable NEL effectors to limit the degree of ETI in mammalian and plant-adapted pathogens. Mechanistically, structural studies reveal that target binding causes the NEL effector to undergo a substantial conformational change, exposing the catalytic site. In addition, the variable length of the LRR domain enables the recognition of a range of different host targets, supporting target diversification (Quezada et al., 2009). Whether NEL effector activity is detected and/or regulated by host proteins remains to be determined, but their unique structural properties represent an opportunity for the development of inhibitors that specifically target the bacterial virulence factors without affecting host E3 ligases.

In contrast to NEL ligases mediating ubiquitination through hijacking of host machinery, *Legionella* effector MavC uses a remarkable E1-independent ubiquitin ligation method to block eukaryotic ubiquitination mediated *via* a specific E2 protein, Ube2N. Although MavC has some structural and functional similarities to the bacterial deamidases Cif from EPEC and CHBP from *Burkholderia pseudomallei*, MavC also contains a unique “insertion” domain which recognizes and interacts with the Ube2N-ubiquitin conjugate (**Figure 2C**) (Yao et al., 2009; Cui et al., 2010; Valleau et al., 2018; Puvar et al., 2020). Rather than exhibiting deamidase activity, MavC catalyzes an intramolecular covalent transglutamination reaction between ubiquitin (Ub) and the host E2 Ube2N, resulting in a γ-glutamyl-ϵ-Lys (Gln40^Ub^-Lys92^Ube2N^) isopeptide crosslink. Functionally, this inactivates the E2-ubiquitin conjugate in the Uev1a:Ube2N-Ub complex, where Uev1a is a non-catalytic partner protein, preventing Lys63-linked poly-ubiquitin chains, and ultimately NF-κB (nuclear factor κ-light-chain-enhancer of activated B cells) activation (Valleau et al., 2018; Puvar et al., 2020). Notably, structural analysis and biochemical functional assays were key in revealing how the deamidase core appended to an insertion domain enabled ubiquitination in a MavC-dependent manner without nucleotide-dependent activation of ubiquitin.

The *Legionella* effector SdeA, part of the SidE effector family, adopts another novel variation of ubiquitination known as phosphoribosyl-linked (PR) ubiquitination. SdeA catalyzes the conjugation of ubiquitin to a target protein on serine residues in an E1 and E2 independent manner (Bhogaraju et al., 2016; Qiu et al., 2016; Akturk et al., 2018; Kim et al., 2018). Structural analysis showed that the mono-ADP-ribosyltransferase (mART) and phosphodiesterase (PDE) domains are the key catalytic domains in SdeA. The mART domain binds to ubiquitin with a novel binding model that is distinct from known eukaryotic ubiquitin-protein interactions and undergoes significant conformational changes in order to ADP-ribosylate arginine-42 of ubiquitin (Ub^R42^) with cofactor NAD^+^. Subsequently, the PDE domain cleaves the phosphodiester bond of ADP-ribosylated ubiquitin, resulting in phosphoribosyl ubiquitin (PR-Ub), which can be linked to the hydroxyl group of serine residues in target substrate proteins (Dong et al., 2018). Interestingly, this non-canonical PR serine ubiquitination in the host cell is tightly regulated and can be reversed by *Legionella* effectors encoding deubiquitinases for PR-linked ubiquitination (DUPs; DupA and DupB) (Shin et al., 2020). Meta-effectors also regulate SidE family member activity. SidJ, a pseudokinase that is activated upon calmodulin binding in the host cell, polyglutamylates SidE ubiquitin ligases, regulating the function of these effectors within the host. Structural analysis convey that the protein kinase fold in SidJ catalyzes the ATP-dependent isopeptide bond formation between the free glutamate amino group and the SidE active site glutamate γ-carboxyl group (Black et al., 2019).

Other effectors also show a high degree of specificity in terms of activity, only becoming activated upon binding to host proteins. *Shigella* T3SS effector OspG, and its homologous effectors NleH1 and NleH2 from EPEC, are atypical serine/threonine kinases that share sequence homology to eukaryotic kinase subdomains I-VII (Zhou et al., 2013). However, they appear to lack other kinase components, including the kinase core and the activation loop. This results in a low or undetectable kinase activity that makes the protein inactive in the bacteria and may prevent non-specific activity in host cells that could initiate ETI. Only upon binding to host ubiquitin, including poly-ubiquitin chains and ubiquitin-conjugated proteins, through hydrophobic interactions mediated by the C-terminal region, is the autophosphorylation and intrinsic ATP hydrolysis activity of OspG stimulated (Zhou et al., 2013). This stimulation enables OspG to phosphorylate host ubiquitin-conjugating enzyme (e.g., UbcH5), which subsequently prevents canonical degradation of phosphorylated inhibitor of NF-κB type α (phospho-IκBα) and TNF-α stimulated NF-κB activation (Kim et al., 2005; Zhou et al., 2013). In contrast, NleH1 and NleH2, which also contain an atypical kinase in the C-terminal domain, autophosphorylate serine and threonine residues in their N-terminal domain independent of ubiquitin binding (Zhou et al., 2013). Autophosphorylation promotes interaction with and phosphorylation of target proteins. The substrate specificities and functional differences may be a result of sequence variation in the N-terminal domains NleH1 and NleH2. NleH1 phosphorylates and inhibits host MAPK (mitogen-activated protein kinase) proteins, ERK1/2 (extracellular signal-regulated kinase 1/2) and p38, to suppress NF-κB activation and apoptosis, whereas NleH2 only inhibits p38 and apoptosis (Zhou et al., 2013; Kralicek et al., 2018). As well as atypical kinases, canonical serine/threonine effector kinases, such as *Yersinia* spp. effector YpkA are also regulated by autophosphorylation. Stimulated YpkA binds and phosphorylates the heterotrimeric G protein complex (Gαq), inhibiting G protein-coupled receptor signaling in the host cell (Navarro et al., 2007; Pha et al., 2014). Overall, it is clear that regulation of effector activity *via* interaction with host proteins and PTM represents a key mechanism by which effector function is tightly regulated within host cells.

### Novel Effector-Mediated Post-Translational Modifications

NEL family E3 ligases and kinase effectors carry out biochemical processes (ubiquitination and phosphorylation respectively) that are found in eukaryotic cells and can therefore be reversed by host cell enzymes. In contrast, other effectors have evolved biochemical activities that appear to mediate irreversible PTMs. For example, the *Shigella* T3SS effector OspF was identified as a phosphothreonine lyase through mass spectrometry of host targets (Li et al., 2007). OspF shares 63% sequence identity with the *Salmonella* T3SS effector SpvC and both proteins catalyze an irreversible phosphate elimination reaction (Mazurkiewicz et al., 2008). Phosphate elimination converts phosphothreonine or phosphoserine to dehydrobutyrine or dehydroalanine respectively and prevents re-phosphorylation of the residue, unlike dephosphorylation. Residues in various MAPKs, including ERK, p38 and JNK, with the dual-phosphorylated pT-X-pY motif, are targeted (Zhu et al., 2007; Mazurkiewicz et al., 2008). This leads to the impairment of MAPK signaling and blocks the activation of pro-inflammatory NF-κB regulated genes and the expression of pro-inflammatory cytokines (Li et al., 2007; Mazurkiewicz et al., 2008). This is likely to be a highly potent method for interference of host cell signaling as the protein cannot be reactivated and instead requires *de novo* protein synthesis. Of the phospholyases described to date, there is a high degree of homology, with the residues required for catalysis fully conserved. However, there is substrate specificity among this effector family which presumably reflects the differing niches of the pathogens that encode them.

Another family of bacterial effectors mediating a variation to known eukaryotic biochemistry is the family of NleB glycosyltransferases. EPEC T3SS effector NleB and the orthologs, SseK1, SseK2 and Ssek3 from *Salmonella*, catalyze the transfer of *N*-acetylglucosamine (GlcNAc) (Gao et al., 2013; Pearson et al., 2013; Li et al., 2013). In the case of NleB, modification of various host death domain-containing proteins, such as FADD, TRADD, and RIPK1, disrupts NF-κB signaling and apoptosis, presumably by preventing host protein dimerization (Pearson et al., 2013; Li et al., 2013). The conserved DXD motif is key in coordinating a metal divalent cation required for the transfer of GlcNAc onto the guanidino group of a target arginine residue (Esposito et al., 2018; Park J. B. et al., 2018). Mechanistically, this is divergent to other known enzymes, which mediate *N*-linked or *O*-linked GlcNAcylation in eukaryotes, with GlcNAc attached to the amide nitrogen in asparagine residues or the hydroxyl oxygen of serine or threonine residues respectively. Structurally, SseK3 displays a classical retaining glycosyltransferases-A (GT-A) Rossman like fold, where substrates are retained until the transfer reaction is complete (Esposito et al., 2018; Newson et al., 2019). Functionally, some substrates of NleB and SseK family members overlap, for example SseK1 modifies FADD and TRADD, whereas SseK3 modifies only TRADD. Together, SseK1, and SseK3 prevent necroptosis of infected macrophages, suggesting some redundancy in effector function (Günster et al., 2017). In addition, SseK3 appears to have evolved to modify small Rab GTPases such as Rab1 (Meng et al., 2020). Intriguingly, during macrophage infection, SseK2 does not appear to show significant arginine-GlcNAcylation at all (Günster et al., 2017). Differences in the surface electrostatic charge distribution between the SseK and NleB family members likely mediates the observed variation in host targets (Günster et al., 2017; Newson et al., 2019). Therefore, as seen with the NEL family of effectors, shared structural and biochemical activities do not always result in functional homology from effectors of different species. This underpins the importance of characterising each bacterial effector in the physiologically relevant context and not relying on the structure-function model to predict the function of any individual effector. In summary, effector-mediated arginine-GlcNAcylation represents a highly potent PTM, irreversible by host cell enzymes. Therefore, as with NEL effectors, inhibitors can be developed to target this unusual and distinct modification, providing a potential alternative to antibiotic therapy that targets virulence factors of the pathogen.

FIC (family of filamentation induced by cyclic adenosine monophosphate) domains, which typically bind ATP and transfer adenosine monophosphate (AMPylation) onto target proteins (Yarbrough et al., 2009), are found in bacterial effectors from diverse species including *Vibrio*, *Legionella*, and *Bartonella* and are also conserved from bacteria to humans (Worby et al., 2009). However, as seen above, evolutionary pressures exerted by the host, drives functional, and biochemical diversification of effectors. In the case of *Legionella* type IV effector AnkX, the FIC domain of AnkX appears unique. Instead of mediating nucleotidyl transferase activity, the FIC domain of AnkX mediates the covalent attachment of a phosphocholine moiety onto a serine residue of host Rab GTPases, including Rab1 and Rab35, modifying Rab function in the host cell (Mukherjee et al., 2011). Similar to AMPylation, the donor molecule for phosphocholination is a nucleotide-based substrate. However, rather than interacting with ATP to transfer the nucleotide moiety, AnkX interacts with cytidine diphosphate (CDP)-choline and transfers the phosphocholine moiety onto hydroxyl-containing residues of target proteins (**Figure 2D**). Structural analysis of AnkX reveals that the orientation of CDP-choline provides an explanation for FIC-motif-mediated transfer of phosphocholine (Campanacci et al., 2013; Ernst et al., 2020). Unexpectedly, the Ankyrin repeats, which normally mediate protein-protein interactions, mediate intramolecular interactions within AnkX. Of note, a second effector from *Legionella*, Lpg0696 (Lem3) has the ability to remove the phosphocholine group from Rab1, restoring the GTPase to its unmodified state (Tan et al., 2011; Goody et al., 2012; Heller et al., 2015). Presumably, this allows for the exquisite control of Rab activity during *Legionella* infection of cells, exploiting an unconventional posttranslational modification that has otherwise only been described for secreted placental polypeptides (Lovell et al., 2007).

This section has described examples of bacterial effectors that mediate covalent modifications with interesting biochemistry. We described effectors that carry out PTMs commonly found in both eukaryotes and bacteria, such as phosphorylation as well as PTMs that, despite only occurring in eukaryotes, have been adopted by bacterial effectors in order to manipulate eukaryotic intracellular signaling, such as ubiquitination. Some of these effectors mediate eukaryotic PTMs *via* non-canonical mechanisms that have not previously been described, such as PR-ubiquitination and transglutamination. Whereas other effectors mediate variations of known chemistry, for example the GlcNAcylation of non-canonical residues and the NEL effectors functioning like eukaryotic E3 ligases. There are also effectors that mediate unconventional PTMs that have not been seen before or are rare in eukaryotes, such as phosphocholination and irreversible phosphate elimination. Together, these studies provide intriguing perspectives and comparisons to traditional eukaryotic-like PTMs. The analysis of novel effector biochemistry also provides fresh and exciting research potential for the development of anti-microbial therapies that target bacteria-specific mechanisms within the host cell.

## Intrinsic Disorder in Bacterial Effectors

Although the function of a protein is generally related to its three-dimensional structure, the classical structure-function paradigm is not applicable to all proteins. A lack of globular structure is found in many proteins, including bacterial effectors. Proteins that are unstructured and flexible with little or no secondary and/or tertiary structure under physiological conditions are referred to as intrinsically disordered proteins (IDPs). IDPs range from fully unstructured to partially unstructured proteins, which contain intrinsically disordered regions (IDRs) (Uversky, 2011). In contrast to ordered protein sequences, IDPs and IDRs have low sequence complexity and contain residues with low mean hydrophobicity and high net charge at neutral pH (Romero et al., 2001; Dyson and Wright, 2005; Vacic et al., 2007b), which leads to intrinsic disorder.

In a study consisting of a large group of structures (16,370 structures) from 5,434 different proteins and from 910 different organisms, only ~7% contained no disorder and only ~25% of structures had >95% of their sequence resolved (Gall et al., 2007). A lack in structural resolution can arise for several reasons, including the presence of transmembrane domains and other factors that impact crystal packing, yet this finding suggests that a large proportion of PDB (protein data bank) structures contain disordered and flexible regions that are not observed in electron density maps. The prevalence of IDRs (in >50 residues) in eukaryotic proteomes is relatively high, with an average of 20%, whereas the IDR abundance is lower in bacterial proteomes (8% on average) and other prokaryotic proteomes (Dunker et al., 2000). In the human genome, cell signaling proteins are particularly enriched with IDRs; with approximately 70% of them predicted to contain long IDRs (Iakoucheva et al., 2002; Marín et al., 2012; Marín et al., 2013). This likely reflects the biological importance of intrinsic disorder in regulating protein-protein interactions for signaling proteins.

**Figure 3:**
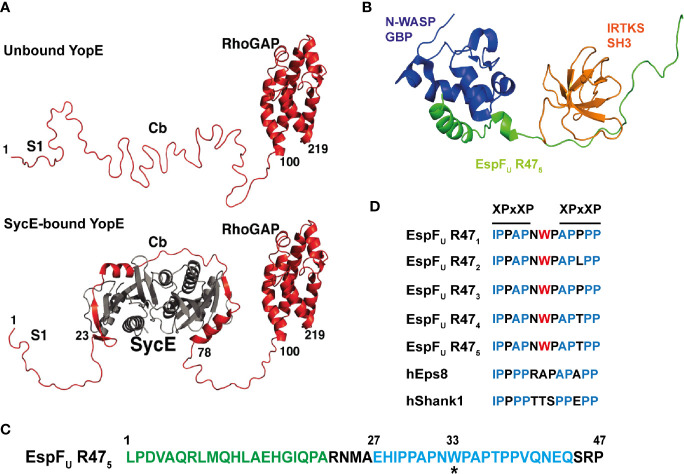
Intrinsic disorder in bacterial effector proteins. **(A)** Crystal structure of *Yersinia* YopE (red) as unbound ‘free’ and bound to the chaperone SycE (grey) in ribbon representation. Functional regions of YopE are labelled: N-terminal secretion signal 1 (S1), chaperone-binding (Cb) and Rho-GAP domain. Intrinsic disorder occurs in the first 100 residues of YopE, which includes the S1 and Cb regions. The chaperone, SycE (gray), binds to the Cb region and cause disorder-to-order conformational change in the Cb region of YopE (taken and adapted from Rodgers et al., 2008). **(B)** Structure of the tri-molecular complex consisting of the GTPase binding domain (GBD) domain of N-WASP, the fifth consecutive 47-residue repeat of EspF_U_ (EspF_U_ R47_5_) and the SH3 domain of IRTKS at the lowest energy conformation in ribbon representation. Structure obtained from NMR spectroscopy. N-WASP GBD, EspF_U_ R47_5_, and IRTKS SH3 are shown and labelled in dark blue, green and orange, respectively (PDB accession number 2LNH from Aitio et al., 2012). **(C)** Amino acid sequence of the fifth repeat of EspF_U_ (EspF_U_ R47_5_). This repeat is one of the highly conserved consecutive 47-residue repeats in EspF_U_. The GBD domain of N-WASP interacts and binds to the N-terminal helix binding region shown in green, and the SH3 domain of IRTKS binds to the C-terminal Proline-rich region shown in blue. The asterisk (*) indicates the tryptophan switch in the linker region (adapted from Aitio et al., 2012). **(D)** Sequence alignment of the linker between the two XPxXP motif in the EspF_U_ repeats and in other IRTKS binding interaction partners, including human Eps8 (hEps8) and human Shank1, showing the tryptophan switch in the linker region. In the XPxXP motif, “X” is a hydrophobic residue and “x” is any residue and P is proline (adapted from Aitio et al., 2012).

Despite the overall low abundance of IDRs in bacterial proteomes, a large number of secreted bacterial effectors are enriched with IDRs. Long disordered regions in the middle and/or in the C-terminal regions are found in roughly 60% of *Salmonella enterica* effectors, 52% of *Pseudomonas syringae* effectors and 71% in *Xanthomonas* subspecies (Marín et al., 2013). This enrichment of IDRs in effectors when compared to the rest of the bacterial proteome suggests the existence of strong selective pressures. For example, structural flexibility is likely to be important for secretion, with disordered regions reducing the need for active unfolding prior to delivery through the narrow T3SS. The poorly described secretion signal for T3SS effectors often represents a disordered region (Samudrala et al., 2009). In many cases, intrinsically disordered N-terminal regions of T3SS effectors have been described to undergo partial folding when bound to a chaperone, as seen for *Yersinia* effector YopE and its chaperone SycE (**Figure 3A**) (Rodgers et al., 2008; Aepfelbacher et al., 2011). This disorder-to-order transition forms a three-dimensional targeting signal that promotes the translocation of YopE through the T3SS (Rodgers et al., 2008).

In addition to effector secretion, the structural flexibility in IDPs/IDRs provides additional advantages. Increased structural flexibility enables IDPs and IDRs to interact with multiple proteins once inside the host cell, adopting various conformations that depend on the structurally divergent interaction partners (Dyson and Wright, 2002; Dyson and Wright, 2005). This is due to the lack of compactness enabling IDRs to expose more surface area per residue, resulting in the exposure of more potential binding sites, at a lower energetic cost to the cell compared to ordered regions of the same residue length (Cortese et al., 2008; Nishikawa and Hatakeyama, 2017). These regions may have evolved to functionally mimic the disordered regions of eukaryotic proteins and support effector-mediated interference with host cell signaling. Indeed, analysis of eukaryotic linear motifs and bacterial motifs show that motif mimicry of eukaryotic motifs is commonly found in bacterial effector proteins (Kumar et al., 2020). Moreover, the flexible nature allows the IDR of proteins to easily access and fit into the catalytic cleft of protein-modifying enzymes for post-translational modification, which enables further regulation of protein-protein interactions and therefore function. For example, the *Helicobacter pylori* type IV secreted effector CagA, contains an intrinsically disordered C-terminal region that acts as a scaffold for multiple interactions with host proteins. Such short linear motifs in the intrinsically disordered C-terminal region, including the Glu-Pro-Ile-Tyr-Ala (EPIYA) motif and the CagA multimerization (CM) motif, are essential for the biological activity of CagA as an oncogenic virulence factor that promotes the transformation of gastric epithelial cells into gastric cancer cells (Nishikawa et al., 2016). Interestingly, the CM motif mimics host substrates in terms of sequence and structure. However, rather than being a substrate, CagA binding actually inhibits the activity of the PAR1 kinase (polarity-regulating serine/threonine kinase in partitioning-defective 1, also known as MARK). This leads to defective cell junctions and polarity of epithelial cells (Saadat et al., 2007; Nešić et al., 2010; Nishikawa et al., 2016). Furthermore, the EPIYA motif undergoes tyrosine phosphorylation by host kinases and this promotes interaction with the Src homology 2 (SH2) domain-containing proteins, such as the pro-oncogenic tyrosine phosphatase SHP2. This induces a conformational change in SHP2 that unlocks the autoinhibitory conformation resulting in deregulated and aberrant SHP2 phosphatase activity, promoting pro-oncogenic mitogenic Ras-ERK signaling and abnormal cell morphology and motility (Higashi et al., 2002; Nishikawa et al., 2016; Hatakeyama, 2017).

In fact, the EPIYA motif is found in IDRs of several bacterial effectors (Hayashi et al., 2013). Each functioning in a tyrosine phosphorylation-dependent manner where the phosphorylated EPIYA or EPIYA-related motifs interact with SH2 domain-containing proteins, causing abnormal and aberrant host cell signaling. Most of these effectors are structurally poorly characterized. However, CD spectroscopy and analytical ultracentrifugation of EPEC effector Tir revealed a monomeric, highly elongated conformation at physiological conditions with a lack of secondary structures. This suggests that Tir is natively unfolded and disordered in solution. Upon phosphorylation of Ser434 and Ser463 by cAMP kinase, Tir undergoes conformational changes that may promote membrane insertion and possibly intermolecular interactions that are required for biological function (Race et al., 2007). Upon tyrosine phosphorylation at the EPIYA-related motif by host kinases, Tir interacts and forms a complex with the SH2 domain containing adaptor protein Nck, promoting actin polymerization (Hayashi et al., 2013).

Furthermore, structural flexibility in IDPs/IDRs may also play a part in protein evolution, potentially providing a selective advantage in comparison to ordered folded protein regions in bacterial effectors. The disordered motifs of CagA are more exposed to the host cell cytoplasm and more prone to sequence polymorphism. These polymorphisms influence the binding affinity to host target proteins and determines the pro-oncogenic degree exhibited by each CagA variant (Nishikawa and Hatakeyama, 2017; Hatakeyama, 2017). Therefore, the disordered nature of IDPs means they are more susceptible to mutational changes as natural selection drives the reduction of molecular disorder, or entropy in thermodynamic terms. This means intrinsically disordered effectors will evolve faster than ordered proteins, with subsequent mutations possibly altering effector interaction partners or enabling the acquisition of new effector functions (Brown et al., 2011; Nishikawa and Hatakeyama, 2017).

Aromatic residues are often involved in protein-protein interactions, but their overall representation is rare in IDPs. However, aromatic residues are found to be enriched in linear motifs or short molecular recognition elements of IDPs. This enables a high degree of specificity to be achieved in a stimulus-dependent manner, and is often observed in proteins involved in signal transduction (Vacic et al., 2007a; Uversky, 2011). Although IDPs typically form weak molecular interactions, the intrinsically disordered EHEC T3SS effector EspF_u_ (also known as TccP) forms a high affinity tri-molecular complex with host proteins N-WASP and insulin receptor tyrosine kinase substrate (IRTKS) (**Figure 3B**). As revealed by NMR spectroscopy, EspF_u_ is a 337-residue IDP that consist of a N-terminal secretion signal followed by highly conserved consecutive 47-residue repeats. Each highly conserved repeat contains the GTPase binding domain (GBD) that interacts with N-WASP and the XPxXP motifs that promote interaction with the Src homology 3 (SH3) domain of IRTKS (**Figure 3C**). Interestingly, there is a tryptophan residue in the linker between the two XPxXP motifs in the EspF_U_ sequence that is absent in known host SH3 interaction partners of IRTKS (**Figure 3D**). This tryptophan appears to have evolved to enable superior binding affinity to outcompete host cellular targets. In this manner, a high affinity tri-molecular complex forms that stimulates actin polymerization for intestinal colonization of EHEC (Aitio et al., 2012). As IDPs can adopt multiple conformations and the conformation constantly changes depending on its interactions and biochemical environment (Marín et al., 2013), it is interesting to postulate that the lack of structure within IDPs enables these bacterial effectors to avoid direct recognition by host inhibitory proteins and the initiation of ETI.

Overall, structural analysis of IDPs or IDRs in proteins is difficult due to the flexible and disordered nature, tendency for degradation, and presence of multiple conformations. Hence, the majority of available structural data on bacterial effectors is limited to structured, ordered, and folded protein regions. Despite this, more work is required as undoubtedly IDRs are important with respect to effector function and therefore their analysis will provide a better understanding of pathogenesis.

## Concluding Remarks and Challenges

Although significant progress has been made over the past two decades, many effectors across diverse pathogens await structural and functional characterization. In different bacterial species, the repertoire of effectors varies in terms of both the number of effectors and the degree to which they have been characterized. For example, 28 out of the 44 identified *Salmonella* T3SS effectors are characterized to a large extent in terms of structure, physiological function and biochemical activity, with only a handful of effectors being completely elusive (Ramos-Morales, 2012; Jennings et al., 2017). A similar degree of characterization has been achieved for EPEC, EHEC and *Shigella*. In contrast, there are many T2SS, T3SS, and T6SS effectors in *Burkholderia pseudomallei* that await validation and characterization (Broek and Stevens, 2017). Work with *B. pseudomallei* requires access to a category III containment laboratory and this may in part explain the poor degree of effector characterization for this species. Perhaps the most striking case is the effector repertoire of *Legionella* species, where more than 18,000 effectors have been identified across the entire genus through genomic analysis. Within this repertoire, 137 different eukaryotic domains were identified with more than 200 effectors containing these eukaryotic-like protein features (Gomez-Valero et al., 2019). This suggests there will be an enormous degree of novel protein domains present among *Legionella* effectors that await characterization.

So, why do so many effectors remain functionally uncharacterized? One significant reason is that primary amino acid sequence is a poor indicator of secondary and tertiary structure and hence biochemical function. For this reason, X-ray crystallography represents an important method for determining the overall tertiary structure and hence the putative biochemical activity of an effector. Yet, even this represents just the start of the road. As described above, a previously unstudied effector may structurally resemble a given eukaryotic enzyme, but actually carry out a modified or an entirely new biochemical function. This makes it difficult to functionally characterize bacterial effectors, for both those with no similarity and even for those where similar effectors have already been studied. Uncovering novel effector functions therefore often requires a combination of structural, proteomic and biochemical studies along with infection work and an open mind. Additional complexities then exist as structural studies of effectors are often hampered due to protein insolubility, cytotoxicity, the presence of intrinsically disordered regions, and the fact that numerous effectors are membrane proteins.

Another important consideration is whether a host protein is required in order for the effector to exist in its active conformation; in this case, it may first be necessary to identify physiologically relevant binding partners prior to acquisition of protein complexes. On the other hand, as bacterial effectors tend to show limited structural homology to known proteins, crystallography of effectors in complex with a host interaction partner of known structure might help resolve the “phase problem” and therefore determination of the structure from the diffraction data. For these reasons, alternative structural and biophysical techniques, such as NMR spectroscopy and CD spectroscopy, could be explored in tandem. Alternatively, an emerging structural technique, cryogenic electron microscopy (cryo-EM), can be used to determine biomolecular structures at near-atomic resolution. Cryo-EM is mainly limited to larger biomolecules and complexes and has been instrumental in solving the structures of many bacterial secretion systems (Kooger et al., 2018; Lunelli et al., 2020; Park D. et al., 2018). However, recent advances show that small proteins of less than 50 kDa can be assembled into large symmetric cage complexes or attached to rigid symmetrical scaffolds for cryo-EM imaging (Liu et al., 2018). However, while cryo-EM represents a promising alternative to X-ray crystallography, there are still potential problems; prior structural knowledge on the protein of interest is required and scaffolds may distort the structure of the protein of interest, particularly disordered regions, resulting in physiologically irrelevant structures.

As the overall number of characterized effectors remains relatively low, it is likely that new effector-mediated biochemistries await discovery. Therefore, these challenges should not deter from continued attempts to structurally and functionally determine effectors from diverse pathogens. This is essential for the continued understanding of how bacterial virulence factors manipulate the host system to promote pathogenesis.

## Author Contributions

Conceptualization: HM and TT. Investigation: HM. Writing—original draft: HM. Reviewing and editing: HM and TT. Supervision: TT. All authors contributed to the article and approved the submitted version.

## Funding

The work of the authors is funded by a BBSRC David Phillips Fellowship (BB/R011834/1) to TLMT.

## Conflict of Interest

The authors declare that the research was conducted in the absence of any commercial or financial relationships that could be construed as a potential conflict of interest.
